# Mortality of Philadelphia for October, November, and December, 1852, Arranged from the Record Kept at the Health Office

**Published:** 1853-02

**Authors:** Wilson Jewell


*Mortality of Philadelphia for October, November, and December,* 1852, *arranged from the Record kept at the Health Office.* By WILSON JEWELL, M. D.

The returns of deaths made to the Health Office, for the fourth quarter of the year 1852, embrace a period of ninety-one days, or thirteen weeks, beginning with the 3d of October and ending with January 1st, 1853.

The number of deaths from all causes, reported during this period, have been 1158 of children under twenty years of age, and 1930 adults; in all, 2088. This shows a falling off of 635, or 23½ per cent. of the mean proportion of deaths for the three previous quarters of the year.

By deducting the Still-Born, the deaths from External Causes, Debility and Old Age, there are left 1759 deaths from actual disease.

The deaths among females were 922; males 1166; showing an excess of 244 of the latter sex.

During the quarter, estimating the population at 409,000, there has been about 1 death to every 196 inhabitants, equal to 23 deaths per day.

Of the whole number of deaths recorded, 930, nearly 47 per cent. were under five years of age; and 129, or 6 per cent. were over seventy years of age.

The deaths from Fevers were 282; exceeding those in the 1st and 2d quarter, and twice as many as those recorded for the 3d quarter of the year. Three fifths of the deaths from Fevers, or 166, were from Scarlatina.

Consumption of the Lungs constitute a majority of all the deaths from diseases of the Organs of Respiration; 271 out of 466, ascribed to these organs, are charged directly to Consumption; whereas, if we include those from "Abscess, Hemorrhage, and Gangrene of the Lungs," with "Disease of Lungs" and "Hectic Fever," we shall still further swell the deaths from this destroying malady.

Those diseases from which the greatest number of deaths have occurred during the quarter, are, Consumption of the Lungs, 271; Scarlet Fever, 166; Convulsions, 88; Inflammation of the Lungs, 86; Croup, 71; Dysentery, 55; Marasmus, 55; Typhus Fever, 53; amounting in all to 845, and constituting very nearly the half of all the deaths recorded from actual disease. Of these, 388 or 45 per ct. were in children under 10 years of age.

TABLE No. I.

*Deaths for the Fourth Quarter of* 1852, *classified.*

| | October. | November. | December. | MALE. O. | MALE. N. | MALE. D. | FEMALE. O. | FEMALE. N. | FEMALE. D. | Total. |
|---|---|---|---|---|---|---|---|---|---|---|
| 1. *Endemic and Contagious Diseases.* Zymotic or Epidemic . . | 148 | 163 | 195 | 84 | 90 | 97 | 64 | 73 | 98 | 506 |
| 2. *Uncertain or general seat.* Sporadic diseases . . . . | 70 | 103 | 126 | 37 | 60 | 70 | 33 | 43 | 56 | 299 |
| 3. The Nervous System . . . | 94 | 87 | 133 | 59 | 54 | 85 | 35 | 33 | 48 | 314 |
| 4. Organs of Respiration . . . | 122 | 157 | 187 | 65 | 88 | 92 | 57 | 69 | 95 | 466 |
| 5. " Circulation . . . | 17 | 16 | 11 | 9 | 7 | 6 | 8 | 9 | 5 | 44 |
| 6. The Digestive Organs . . . | 36 | 43 | 49 | 23 | 22 | 26 | 13 | 21 | 23 | 128 |
| 7. The Urinary Organs . . . | 2 | 3 | 1 | 2 | 3 | 1 | | | | 6 |
| 8. The Organs of Generation . . | 1 | 3 | 5 | 1 | | | | 3 | 5 | 9 |
| 9. " Locomotion . . | 3 | 3 | 5 | 2 | 1 | 2 | 1 | 2 | 3 | 11 |
| 10. The Integumentary System . . | 1 | 2 | 1 | 1 | 1 | 1 | | 1 | | 4 |
| 11. Old Age . . . . . . | 9 | 8 | 15 | 4 | 5 | 1 | 5 | 3 | 14 | 32 |
| 12. External Causes . . . . | 22 | 23 | 25 | 16 | 13 | 19 | 6 | 10 | 6 | 70 |
| Still Born . . . . . | 33 | 41 | 56 | 20 | 25 | 35 | 13 | 16 | 21 | 130 |
| Unknown . . . . . | 22 | 25 | 22 | 11 | 17 | 11 | 11 | 8 | 11 | 69 |
| | 580 | 677 | 831 | 334 | 386 | 446 | 246 | 291 | 385 | 2088 |

TABLE No. II.

1. *Endemic and Contagious Diseases—Zymotic or Epidemic.*

| | Male. | Female. | Under 1 yr. | 1 to 2. | 2 to 5. | 5 to 10. | 10 to 15. | 15 to 20. | 20 to 30. | 30 to 40. | 40 to 50. | 50 to 60. | 60 to 70. | 70 to 80. | 80 to 90. | 90 to 100. | 100 to 110. | Total. |
|---|---|---|---|---|---|---|---|---|---|---|---|---|---|---|---|---|---|---|
| Aphthæ . . . | 1 | 1 | 2 | | | | | | | | | | | | | | | 2 |
| Cholera Infantum . | 5 | 4 | 4 | 2 | 3 | | | | | | | | | | | | | 9 |
| " Morbus . | 16 | 8 | | | | | 2 | | 8 | 5 | 3 | 2 | 2 | 1 | 1 | | | 24 |
| Croup . . . | 34 | 37 | 12 | 15 | 36 | 7 | 1 | | | | | | | | | | | 71 |
| Diarrhœa . . . | 11 | 17 | 8 | 7 | 2 | | | | 3 | | | 2 | 4 | | 2 | | | 28 |
| Dysentery . . | 36 | 19 | 5 | 11 | 11 | 4 | | 3 | 6 | 5 | 5 | 2 | 2 | 1 | | | | 55 |
| Erysipelas . . | 6 | 4 | 4 | 1 | | | | | | 3 | | 1 | 1 | | | | | 10 |
| Fever . . . | 2 | 5 | 1 | | | | | 2 | | 1 | 1 | 1 | | | | 1 | | 7 |
| " Bilious . . | 4 | | | | 1 | | | | 3 | | | | | | | | | 4 |
| " Congestive . | 2 | 2 | | | 3 | | | | | | | | 1 | | | | | 4 |
| " Intermittent . | 1 | | | | | 1 | | | | | | | | | | | | 1 |
| " Remittent . | 2 | 3 | 1 | | 1 | | 1 | | | 1 | 1 | | | | | | | 5 |
| " Scarlet . . | 90 | 76 | 16 | 21 | 76 | 49 | 2 | 1 | 1 | | | | | | | | | 166 |
| " Synocha . . | 1 | | | | | | | | 1 | | | | | | | | | 1 |
| " Typhus . . | 22 | 31 | | | 2 | 1 | 1 | 5 | 17 | 6 | 6 | 10 | 5 | | | | | 53 |
| " Typhoid . . | 26 | 15 | 1 | 1 | 4 | | 1 | 5 | 13 | 8 | 5 | 2 | | 1 | | | | 41 |
| Hooping Cough . . | 2 | 3 | 3 | | 2 | | | | | | | | | | | | | 5 |
| Influenza . . . | 1 | 2 | | | | | | | | | | 1 | 1 | 1 | | | | 3 |
| Measles . . . | 2 | 1 | 2 | | 1 | | | | | | | | | | | | | 3 |
| Small Pox. . . | 7 | 6 | 4 | 2 | 5 | 1 | | | 1 | | | | | | | | | 13 |
| Syphilis . . . | | 1 | | | | | | | | 1 | | | | | | | | 1 |
| | 271 | 235 | 63 | 60 | 147 | 63 | 8 | 16 | 53 | 30 | 21 | 21 | 16 | 4 | 3 | 1 | | 506 |

2. *Uncertain or General Seat.—Sporadic Diseases.*

| | Male. | Female. | Under 1 yr. | 1 to 2. | 2 to 5. | 5 to 10. | 10 to 15. | 15 to 20. | 20 to 30. | 30 to 40. | 40 to 50. | 50 to 60. | 60 to 70. | 70 to 80. | 80 to 90. | 90 to 100. | 100 to 110. | Total. |
|---|---|---|---|---|---|---|---|---|---|---|---|---|---|---|---|---|---|---|
| Abscess Psoas . . | | 1 | | | | | | | | 1 | | | | | | | | 1 |
| Angina . . . | | 3 | 1 | | 1 | | | 1 | | | | | | | | | | 3 |
| Cachexia . . . | 1 | 2 | 1 | | | | | | | 1 | 1 | | | | | | | 3 |
| Cancer . . . | 1 | 4 | | | | | | | | 1 | 1 | 1 | 1 | 1 | | | | 5 |
| " Breast . . | | 1 | | | | | | | | | | | | | 1 | | | 1 |
| " Throat . . | 1 | | | | | | | | 1 | | | | | | | | | 1 |
| Cyanosis . . . | 4 | 2 | 4 | 1 | 1 | | | | | | | | | | | | | 6 |
| Debility . . . | 66 | 31 | 38 | 1 | 1 | | | | 2 | 5 | 11 | 6 | 6 | 16 | 9 | 2 | | 97 |
| Disease of Mesen. Glands | | 1 | 1 | | | | | | | | | | | | | | | 1 |
| Disease of Throat . | 1 | | | | | | | | | | | | 1 | | | | | 1 |
| Dropsy . . . | 23 | 14 | | 3 | 5 | 1 | 1 | 2 | 2 | 7 | 6 | 2 | 6 | 2 | | | | 37 |
| Fungus Hematodes . | 1 | | | | | | | | | | | 1 | | | | | | 1 |
| Gangrene . . . | 1 | 3 | | | 1 | | | | | | | 1 | | 1 | 1 | | | 4 |
| Gout in Stomach . . | 1 | | | | | | | | | | | | 1 | | | | | 1 |
| Hemorrhage . . | 3 | 5 | 2 | | | | | | 2 | 2 | 1 | | | 1 | | | | 8 |
| Inanition . . . | 8 | 6 | 11 | | | | | | | 1 | | | 1 | | 1 | | | 14 |
| Inflammation . . | 1 | 4 | | 2 | | | | | | 1 | | 1 | | 1 | | | | 5 |
| " of Neck . . | 1 | | 1 | | | | | | | | | | | | | | | 1 |
| Malformation . . | 5 | 1 | 5 | | | | 1 | | | | | | | | | | | 6 |
| Marasmus . . . | 29 | 26 | 27 | 12 | 8 | 1 | 2 | 1 | 1 | 1 | | | | 1 | 1 | | | 55 |
| Mortification . . | | 1 | | | | | | | | | | | | 1 | | | | 1 |
| Ptyalism . . . | | 1 | | | | | | | | 1 | | | | | | | | 1 |
| Scirrhus . . . | | 2 | | | | | | | | | 1 | | | 1 | | | | 2 |
| Scrofula . . . | 17 | 9 | 3 | 3 | 4 | 2 | 7 | 3 | | | 2 | 1 | 1 | | | | | 26 |
| Tabes Mesenterica . | 3 | 9 | 3 | 1 | 5 | | 1 | | 1 | 1 | | | | | | | | 12 |
| Tumours . . . | | 2 | | | | | | | | | 1 | | 1 | | | | | 2 |
| " of Neck . . | | 1 | 1 | | | | | | | | | | | | | | | 1 |
| Ulceration of Throat . | | 3 | 1 | 1 | 1 | | | | | | | | | | | | | 3 |
| | 167 | 132 | 99 | 24 | 27 | 4 | 12 | 7 | 9 | 22 | 24 | 13 | 18 | 25 | 13 | 2 | | 299 |

3. *Of the Nervous System.*

| | Male. | Female. | Under 1 yr. | 1 to 2. | 2 to 5. | 5 to 10. | 10 to 15. | 15 to 20. | 20 to 30. | 30 to 40. | 40 to 50. | 50 to 60. | 60 to 70. | 70 to 80. | 80 to 90. | 90 to 100. | 100 to 110. | Total. |
|---|---|---|---|---|---|---|---|---|---|---|---|---|---|---|---|---|---|---|
| Abscess of Brain . . | 1 | | | | | | | 1 | | | | | | | | | | 1 |
| Apoplexy . . . | 16 | 8 | | | | 1 | | | 1 | 2 | 4 | 4 | 7 | 5 | | | | 24 |
| Asthma Thymic . . | 1 | | 1 | | | | | | | | | | | | | | | 1 |
| Cerebral Disease . | 1 | | | | | | | | | | | 1 | | | | | | 1 |
| " Fever . . | 1 | | | 1 | | | | | | | | | | | | | | 1 |
| Compression of Brain . | 1 | 1 | | | | | | | 1 | | 1 | | | | | | | 2 |
| Concussion " . | 1 | | | | | | | | | 1 | | | | | | | | 1 |
| Congestion " . | 12 | 7 | 5 | 1 | 2 | 2 | | | 2 | 2 | | | 4 | 1 | | | | 19 |
| Convulsions . . | 57 | 31 | 51 | 16 | 15 | 2 | 1 | 1 | | 1 | 1 | | | | | | | 88 |
| Cramp . . . | 1 | | | 1 | | | | | | | | | | | | | | 1 |
| Disease of Brain . . | 14 | 9 | 9 | 2 | 3 | 1 | 3 | | 1 | 3 | | | | 1 | | | | 23 |
| Dropsy " . . | 19 | 13 | 13 | 8 | 6 | 3 | 1 | | 1 | | | | | | | | | 32 |
| Effusion " . . | 10 | 10 | 2 | 2 | 6 | 1 | | 1 | | 2 | 3 | 3 | | | | | | 20 |
| Epilepsy . . . | 3 | 3 | | | | | 1 | | 2 | | 1 | 1 | | 1 | | | | 6 |
| Hemorrhage from Ear . | | 1 | 1 | | | | | | | | | | | | | | | 1 |
| Inflammation of Brain . | 27 | 18 | 9 | 5 | 15 | 3 | 1 | 3 | 5 | 3 | 1 | | | | | | | 45 |
| Injury of Brain . . | 1 | | | | | | | | | | | | | | 1 | | | 1 |
| Mania . . . | | 2 | | | | | | | 1 | 1 | | | | | | | | 2 |
| " a potu . . | 12 | 3 | | | | | | | | 6 | 4 | 4 | | 1 | | | | 15 |
| Neuralgia . . . | 1 | | | | | | | | | | | | 1 | | | | | 1 |
| Palsy . . . . | 9 | 8 | 1 | | | 1 | | | 2 | | 1 | 2 | 4 | 4 | 2 | | | 17 |
| Softening of Brain . | 7 | 1 | | | 1 | | | | 1 | 2 | 3 | 1 | | | | | | 8 |
| Tetanus . . . | 2 | 1 | | | | 1 | | | 1 | | | 1 | | | | | | 3 |
| Trismus . . . | 1 | | 1 | | | | | | | | | | | | | | | 1 |
| | 981 | 116 | 93 | 36 | 48 | 15 | 7 | 6 | 18 | 23 | 19 | 17 | 16 | 13 | 3 | | | 314 |

### 4. *Organs of Respiration.*

| | Male. | Female. | Under 1 yr. | 1 to 2. | 2 to 5. | 5 to 10. | 10 to 15. | 15 to 20. | 20 to 30. | 30 to 40. | 40 to 50. | 50 to 60. | 60 to 70. | 70 to 80. | 80 to 90. | 90 to 100. | 100 to 110. | Total. |
|---|---|---|---|---|---|---|---|---|---|---|---|---|---|---|---|---|---|---|
| Abscess of Lungs . | 3 | 1 | | | | | | 2 | | 1 | | 1 | | | | | | 4 |
| Asthma . . . | 1 | 1 | | 1 | | | | | | | | | | 1 | | | | 2 |
| Congestion of Lungs . | 4 | 9 | 6 | | 1 | 1 | 1 | 1 | 1 | | | | 2 | | | | | 13 |
| Consumption " . | 134 | 137 | 5 | 7 | 3 | 6 | 4 | 19 | 80 | 57 | 39 | 33 | 11 | 7 | | | | 271 |
| " Laryngeal . | | 1 | | | | | | | | | | 1 | | | | | | 1 |
| Disease of Lungs . | 4 | 1 | 1 | | | 1 | | 1 | 1 | | 1 | | | | | | | 5 |
| " Chest . . | 3 | 1 | 1 | 1 | 1 | | | | | | | 1 | | | | | | 4 |
| Dropsy of Chest . . | 3 | 2 | | 2 | | | | | | 1 | 1 | | 1 | | | | | 5 |
| Effusion " . . | 2 | 1 | | 1 | | 1 | | | | | | | | 1 | | | | 3 |
| " Lungs . . | 1 | 1 | | | | | | | | | | 1 | | | 1 | | | 2 |
| Fever, Hectic . . | 1 | | | | 1 | | | | | | | | | | | | | 1 |
| Gangrene of Lungs . | 1 | | | | | | | | | | | | 1 | | | | | 1 |
| Hemorrhage " . . | 4 | 3 | | | | | | | 3 | 1 | 2 | 1 | | | | | | 7 |
| Inflammation of Bronchiæ | 23 | 18 | 24 | 3 | 4 | 3 | 1 | | 2 | 2 | | | 1 | 1 | | | | 41 |
| " Chest . | 4 | | 1 | 1 | 2 | | | | | | | | | | | | | 4 |
| " Larynx . | 3 | 4 | 1 | | 4 | 1 | | | 1 | | | | | | | | | 7 |
| " Lungs . | 50 | 36 | 17 | 12 | 10 | 2 | 2 | 3 | 16 | 3 | 8 | 5 | 5 | 1 | 2 | | | 86 |
| " Pleura . | | 2 | | | | | 1 | | | 1 | | | | | | | | 2 |
| " Throat . | 1 | 1 | | 1 | | 1 | | | | | | | | | | | | 2 |
| Tuberculosis . . | 3 | 2 | 1 | 1 | | | | | 3 | | | | | | | | | 5 |
| | 245 | 122 | 57 | 30 | 26 | 16 | 9 | 26 | 107 | 66 | 51 | 43 | 21 | 11 | 3 | | | 466 |

### 5. *Organs of Circulation.*

| | Male. | Female. | Under 1 yr. | 1 to 2. | 2 to 5. | 5 to 10. | 10 to 15. | 15 to 20. | 20 to 30. | 30 to 40. | 40 to 50. | 50 to 60. | 60 to 70. | 70 to 80. | 80 to 90. | 90 to 100. | 100 to 110. | Total. |
|---|---|---|---|---|---|---|---|---|---|---|---|---|---|---|---|---|---|---|
| Anæmia . . . | 1 | 1 | 1 | | | | | | | 1 | | | | | | | | 2 |
| Angina Pectoris . | | 1 | | | | | | | | | | | | 1 | | | | 1 |
| Aneurism of Aorta . | 2 | | | | | | | | | | 2 | | | | | | | 2 |
| Disease of Heart . . | 15 | 13 | 3 | | 1 | 3 | | 2 | 3 | 1 | 5 | 4 | 4 | 2 | | | | 28 |
| Dropsy " . . | 1 | | | | | | | | 1 | | | | | | | | | 1 |
| Effusion " . . | 1 | | | | | | | | | | | | | 1 | | | | 1 |
| Enlargement of Heart . | | 2 | | | | | | 1 | | 1 | | | | | | | | 2 |
| Inflammation " . | 1 | 4 | | 1 | | | 1 | | 1 | 1 | | 1 | | | | | | 5 |
| Malformation " . | 1 | 1 | 1 | 1 | | | | | | | | | | | | | | 2 |
| | 22 | 22 | 5 | 2 | 1 | 3 | 1 | 3 | 5 | 4 | 7 | 5 | 4 | 4 | | | | 44 |

6. *Digestive Organs.*

| | Male. | Female. | Under 1 yr. | 1 to 2. | 2 to 5. | 5 to 10. | 10 to 15. | 15 to 20. | 20 to 30. | 30 to 40. | 40 to 50. | 50 to 60. | 60 to 70. | 70 to 80. | 80 to 90. | 90 to 100. | 100 to 110. | Total. |
|---|---|---|---|---|---|---|---|---|---|---|---|---|---|---|---|---|---|---|
| Abdominal Dropsy . | 7 | 3 | | | | | | | 1 | | 2 | 3 | 2 | 2 | | | | 10 |
| Abscess of Liver . . | 2 | 1 | | | | 1 | | | 1 | | 1 | | | | | | | 3 |
| " of Stomach . | 1 | | | | | | | | | | | 1 | | | | | | 1 |
| Cancer of " . . | 1 | 1 | | | | | | | | 1 | 1 | | | | | | | 2 |
| Cancrum Oris . . | 3 | 4 | 2 | | 5 | | | | | | | | | | | | | 7 |
| Cirrhosis of Liver . | | 1 | | | | | | | 1 | | | | | | | | | 1 |
| Disease of Bowels . | 1 | 3 | 3 | | | | | | | | | | | 1 | | | | 4 |
| " Liver . . | 3 | 3 | | | | | | 1 | 1 | 2 | 1 | | | 1 | | | | 6 |
| " Stomach . | | 2 | | | | | | | | 1 | | | | 1 | | | | 2 |
| Dyspepsia . . . | 1 | | | | | | | | | | | 1 | | | | | | 1 |
| Hemorrhage of Bowels | 2 | 1 | | | | | 1 | | 2 | | | | | | | | | 3 |
| " Stomach | 1 | 2 | 1 | | | | | | | 1 | | | 1 | | | | | 3 |
| Hernia, strangulated . | | 1 | | | | | | | | | | 1 | | | | | | 1 |
| Inflam. of Liver . | 3 | 3 | | | | | | | 1 | 1 | 1 | 1 | 2 | | | | | 6 |
| " Peritoneum . | 7 | 6 | 1 | | 1 | | | | 5 | 4 | | | 1 | | 1 | | | 13 |
| " Pharynx . | 1 | | 1 | | | | | | | | | | | | | | | 1 |
| " Stomach . | | 2 | | | | | | | | 1 | | | 1 | | | | | 2 |
| Inflam. Stomach & Bowels | 32 | 16 | 8 | 2 | 5 | 5 | 1 | 2 | 4 | 7 | 6 | 5 | 1 | 2 | | | | 48 |
| Jaundice . . . | 2 | 2 | 2 | | | | | | | 1 | | | 1 | | | | | 4 |
| Obstruction of Bowels . | 1 | 1 | | | | | | | | 1 | | 1 | | | | | | 2 |
| Perforation of Bowels . | | 1 | | | | | | | | | 1 | | | | | | | 1 |
| Stricture of Colon . | 1 | | | | | | | | | | | | 1 | | | | | 1 |
| Teething . . . | 1 | 1 | 1 | 1 | | | | | | | | | | | | | | 2 |
| Ulceration of Bowels . | 1 | 2 | | | | | | | 2 | | | | 1 | | | | | 3 |
| Worms . . . | | 1 | | | 1 | | | | | | | | | | | | | 1 |
| | 71 | 57 | 19 | 3 | 12 | 6 | 2 | 3 | 18 | 20 | 13 | 13 | 11 | 7 | 1 | | | 128 |

7. *The Urinary Organs.*

| | Male. | Female. | Under 1 yr. | 1 to 2. | 2 to 5. | 5 to 10. | 10 to 15. | 15 to 20. | 20 to 30. | 30 to 40. | 40 to 50. | 50 to 60. | 60 to 70. | 70 to 80. | 80 to 90. | 90 to 100. | 100 to 110. | Total. |
|---|---|---|---|---|---|---|---|---|---|---|---|---|---|---|---|---|---|---|
| Disease of Kidneys . | 2 | | | | | | | | 1 | 1 | | | | | | | | 2 |
| " " and Bladder | 1 | | | | | | | | | | | | | 1 | | | | 1 |
| Inflam. of Bladder . | 1 | | | | | | | | | | | | | | 1 | | | 1 |
| " of Kidneys . | 1 | | | | | 1 | | | | | | | | | | | | 1 |
| Urinary Calculus . | 1 | | | | | | | | | | | | 1 | | | | | 1 |
| | 6 | | | | | 1 | | | 1 | 1 | | | 1 | 1 | 1 | | | 6 |

8. *Organs of Generation.*

| | Male. | Female. | Under 1 yr. | 1 to 2. | 2 to 5. | 5 to 10. | 10 to 15. | 15 to 20. | 20 to 30. | 30 to 40. | 40 to 50. | 50 to 60. | 60 to 70. | 70 to 80. | 80 to 90. | 90 to 100. | 100 to 110. | Total. |
|---|---|---|---|---|---|---|---|---|---|---|---|---|---|---|---|---|---|---|
| Enlargemt. Prostate Gland | 1 | | | | | | | | | 1 | | | | | | | | 1 |
| Puerperal Fever . . | | 3 | | | | | | | 2 | 1 | | | | | | | | 3 |
| " Inflam. . | | 1 | | | | | | | | 1 | | | | | | | | 1 |
| Hemorrhage from Uterus | | 1 | | | | | | | 1 | | | | | | | | | 1 |
| Inflammation " | | 1 | | | | | | | | 1 | | | | | | | | 1 |
| Cancer of Uterus . . | | 1 | | | | | | | | | 1 | | | | | | | 1 |
| Phlegmasia Dolens . | | 1 | | | | | | | 1 | | | | | | | | | 1 |
| | 1 | 8 | | | | | | | 4 | 4 | 1 | | | | | | | 9 |

9. *Organs of Locomotion.*

| | Male. | Female. | Under 1 yr. | 1 to 2. | 2 to 5. | 5 to 10. | 10 to 15. | 15 to 20. | 20 to 30. | 30 to 40. | 40 to 50. | 50 to 60. | 60 to 70. | 70 to 80. | 80 to 90. | 90 to 100. | 100 to 110. | Total. |
|---|---|---|---|---|---|---|---|---|---|---|---|---|---|---|---|---|---|---|
| Caries . . . | 1 | 1 | | | | | | | | | 1 | | 1 | | | | | 2 |
| Hip Disease . . | 1 | 1 | | | 1 | | | | 1 | | | | | | | | | 2 |
| Disease of Spine . | | 1 | | | | | | | | 1 | | | | | | | | 1 |
| " Leg . . | 1 | | | | | | | 1 | | | | | | | | | | 1 |
| Cancer " . . | | 1 | | | | | | | | 1 | | | | | | | | 1 |
| Fracture of Spine . | 1 | | | | | | | | 1 | | | | | | | | | 1 |
| Atrophy Spinal Marrow | 1 | | | | | | | | | | | 1 | | | | | | 1 |
| Rheumatism . . | | 1 | | | | | | | | | | | | 1 | | | | 1 |
| Spina Bifida . . | | 1 | 1 | | | | | | | | | | | | | | | 1 |
| | 5 | 6 | 1 | | 1 | | | 1 | 2 | 2 | 1 | 1 | 1 | 1 | | | | 11 |

10. *The Integumentary System.*

| | Male. | Female. | Under 1 yr. | 1 to 2. | 2 to 5. | 5 to 10. | 10 to 15. | 15 to 20. | 20 to 30. | 30 to 40. | 40 to 50. | 50 to 60. | 60 to 70. | 70 to 80. | 80 to 90. | 90 to 100. | 100 to 110. | Total. |
|---|---|---|---|---|---|---|---|---|---|---|---|---|---|---|---|---|---|---|
| Purpura Hemorrhagica | 3 | 1 | 1 | 1 | 1 | | | | 1 | | | | | | | | | 4 |

11. *Old Age.*

| | Male. | Female. | Under 1 yr. | 1 to 2. | 2 to 5. | 5 to 10. | 10 to 15. | 15 to 20. | 20 to 30. | 30 to 40. | 40 to 50. | 50 to 60. | 60 to 70. | 70 to 80. | 80 to 90. | 90 to 100. | 100 to 110. | Total. |
|---|---|---|---|---|---|---|---|---|---|---|---|---|---|---|---|---|---|---|
| Old Age . . . | 10 | 22 | | | | | | | | | | | 3 | 9 | 12 | 6 | 2 | 32 |

12. *From External Causes.*

| | Male. | Female. | Under 1 yr. | 1 to 2. | 2 to 5. | 5 to 10. | 10 to 15. | 15 to 20. | 20 to 30. | 30 to 40. | 40 to 50. | 50 to 60. | 60 to 70. | 70 to 80. | 80 to 90. | 90 to 100. | 100 to 110. | Total. |
|---|---|---|---|---|---|---|---|---|---|---|---|---|---|---|---|---|---|---|
| Asphyxia . . . | 5 | 5 | 8 | 2 | | | | | | | | | | | | | | 10 |
| Burns and Scalds . . | 4 | 6 | 1 | 3 | 3 | 2 | | | | 1 | | | | | | | | 10 |
| Casualties . . . | 18 | 4 | 5 | | 1 | 1 | | | 4 | 3 | 3 | 5 | | | | | | 22 |
| Drowned . . . | 9 | 2 | | | | 1 | 1 | 2 | 2 | 3 | 1 | 1 | | | | | | 11 |
| Intemperance . . | 5 | 2 | | | | | | | 1 | 2 | 3 | 1 | | | | | | 7 |
| Suffocation . . . | 2 | 3 | 2 | | | | | | | 1 | | | 2 | | | | | 5 |
| Suicide . . . | 3 | | | | | | | | | 1 | 2 | | | | | | | 3 |
| Violence . . . | 1 | | | | | | | 1 | | | | | | | | | | 1 |
| Exposure . . . | 1 | | | | | | | | | 1 | | | | | | | | 1 |
| | 48 | 22 | 16 | 5 | 4 | 4 | 1 | 3 | 7 | 12 | 9 | 7 | 2 | | | | | 70 |

| | Male. | Female. | Under 1 yr. | 1 to 2. | 2 to 5. | 5 to 10. | 10 to 15. | 15 to 20. | 20 to 30. | 30 to 40. | 40 to 50. | 50 to 60. | 60 to 70. | 70 to 80. | 80 to 90. | 90 to 100. | 100 to 110. | Total. |
|---|---|---|---|---|---|---|---|---|---|---|---|---|---|---|---|---|---|---|
| Unknown . . . | 39 | 30 | 20 | 4 | 3 | | 1 | 1 | 4 | 10 | 6 | 7 | 6 | 4 | | 3 | | 69 |
| Still Born . . . | 80 | 50 | 130 | | | | | | | | | | | | | | | 130 |

### TABLE NO. 3.

*Deaths for the Fourth quarter, at fifteen distinct periods of life.*

| | |
|---|---|
| Under 1 year, . . . . . . | 374 |
| 1 to 2 . . . . . . | 165 |
| 2 to 5 . . . . . . | 270 |
| 5 to 10 . . . . . . | 112 |
| 10 to 15 . . . . . . | 41 |
| 15 to 20 . . . . . . | 66 |
| 20 to 30 . . . . . . | 229 |
| 30 to 40 . . . . . . | 194 |
| 40 to 50 . . . . . . | 152 |
| 50 to 60 . . . . . . | 127 |
| 60 to 70 . . . . . . | 99 |
| 70 to 80 . . . . . . | 79 |
| 80 to 90 . . . . . . | 36 |
| 90 to 100 . . . . . . | 12 |
| 100 to 110 . . . . . . | 2 |
| | 1958 |
| Still Born . . . . . . . | 130 |
| Total, | 2088 |

Included in the above Tables, were 193 from the Blockley Almshouse; 171 people of color, and 18 from the country; as follows:

| | Almshouse. | Blacks. | Country. |
|---|---|---|---|
| October, . . . . | 42 | 48 | 9 |
| November, . . . . . | 66 | 58 | 5 |
| December, . . . . | 85 | 65 | 4 |
| Total, | 193 | 171 | 18 |

SUMMARY FOR THE WHOLE YEAR.

The quarterly tables presented in this Journal for the year 1852, exhibit, in those marked No. 1, the number of deaths, with their causes, for each month, grouped or classified in accordance with the system approved by the American Medical Association. They also furnish the sexes in which the deaths took place.

The tables marked No. 2, show in their classified relations, not only the number of deaths, but the names of the diseases causing death, as given in the certificates returned to the Board of Health. They are arranged under fifteen distinct periods of life.

The tables, No. 3, furnish the aggregate of deaths at fifteen distinct periods of life.

There are also appended, the deaths which took place during each quarterly period, at the Blockley Almshouse; the number of deaths among the colored population, as well as the number brought from the country to be interred in the city.

The following table exhibits the deaths for the year 1852, in each sex, under the several classes of disease:

| | Male. | Female. | Total. |
|---|---|---|---|
| 1. *Endemic and Contagious diseases.* Zymotic or Epidemic, | 1398 | 1387 | 2785 |
| 2. *Uncertain or General seat.* Sporadic diseases, | 698 | 575 | 1273 |
| 4. The Nervous System, | 957 | 712 | 1669 |
| 3. Organs of Respiration, | 1147 | 1025 | 2172 |
| 5. " " Circulation, | 114 | 105 | 219 |
| 6. Digestive Organs, | 313 | 314 | 627 |
| 7. Urinary Organs, | 28 | 4 | 32 |
| 8. Generative Organs, | 8 | 104 | 112 |
| 9. Locomotive Organs, | 26 | 20 | 46 |
| 10. Integumentary System, | 9 | 9 | 18 |
| 11. Old Age, | 57 | 135 | 192 |
| 12. External Causes, | 240 | 82 | 322 |
| Still Born, | 293 | 223 | 516 |
| Unknown, | 149 | 126 | 275 |
| | 5437 | 4821 | 10258 |

In accordance with these tables, the aggregate of deaths from all causes for the year was 10,258, an excess of 1387, or

13 per cent. over those for 1851. This aggregate furnishes one death to every 39 8-10ths of a population of 409,000.

Included in the above total were 913 from the Blockley almshouse, 832 blacks and 108 from the country, in all 1853.

The highest number of deaths in any one month, occurred in January, amounting to 1037, making 33½ deaths per day. The lowest number was in October, viz. 580; equal to 18¾ deaths per day. The mean number of deaths per day, throughout the year was 28.

Of the whole number of deaths, 5049, or 49 per cent., were among children under five years of age. Of this great excess of mortality in infancy, 2800, or 27½ per cent. occurred before the termination of the first year of life. In this calculation we have included the "still-born," which amounted to 516. How many of these were premature, or how many had reached the full period of gestation, we have no means of ascertaining. This information would be valuable in order to show the risks of life encountered during the eventful period of parturition.

Of the deaths from all causes, exclusive of still-born, 590, or 6 per cent., were beyond 70 years of age. Of these, 188 were over 80; 38 were over 90, and 7 were between 100 and 110 years of age.

The most prevalent diseases in the community, and the number of deaths therefrom during the year, have been as follows: Consumption of the Lungs, 1204; Dysentery, 558; Convulsions, 499; Inflammation of the Lungs, 444; Scarlet Fever, 433; Small Pox, 426; Marasmus, 354; Debility, 345; Cholera Infantum, 329; Inflammation of the Brain, 258; Dropsy of the Brain, 247; Inflammation of the Bronchiæ, 208; Croup, 208. In all, 5513, constituting a majority of the whole number of deaths recorded.

In the aggregate of deaths we find the preponderance on the side of the males. While those among females amounted to 4821, the male deaths were 5437, an excess of 616, or about 13 per cent., over the females.

Among the "Still-Born" the excess was 30 per cent. on the side of the males. From "External Causes," there were three males to one female. While among the deaths from "Old Age" there were two females to one male.

The diseases under the head of Zymotic, have contributed a very large proportion towards making up the deaths for the year, viz. 2785, or 28½ per cent. of the whole number. The Sporadic diseases caused about 13 per cent. The diseases of the Nervous System have contributed 1669, or 17 per cent. Those of the Organs of Respiration 2172, or nearly 22 per ct. These calculations do not include the "Still-Born, 516."

The deaths, specified in the record as Consumption of the Lungs, number 1204: this gives one out of every 8½ deaths during the year, or nearly 12 per cent. There is an increase of mortality from this disease over the year 1851, of 35 per cent. The deaths from Consumption for 1852, to population, is as one to 90½.

The following table presents a comparison between the diseases which have been most fatal, hence most prevalent, for five consecutive years:

| | 1848 | 1849 | 1850 | 1851 | 1852 |
|---|---|---|---|---|---|
| Cholera Infantum, | 454 | 582 | 505 | 397 | 329 |
| Consumption of the Lungs, | 965 | 939 | 907 | 881 | 1204 |
| Congestion of the Brain, | 85 | 91 | 97 | 130 | 120 |
| Convulsions, | 401 | 415 | 444 | 479 | 499 |
| Croup, | 177 | 130 | 143 | 180 | 208 |
| Debility, | 145 | 200 | 201 | 260 | 345 |
| Diarrhœa, | 122 | 225 | 208 | 157 | 156 |
| Dropsy of the Brain, | 220 | 237 | 283 | 245 | 247 |
| Dysentery, | 315 | 578 | 421 | 401 | 558 |
| Scarlet Fever, | 172 | 242 | 439 | 400 | 433 |
| Inflammation of the Brain, | 186 | 198 | 218 | 202 | 258 |
| " " Bronchiæ, | 172 | 169 | 191 | 175 | 208 |
| " " Lungs, | 265 | 273 | 352 | 352 | 444 |
| Marasmus, | 237 | 264 | 217 | 255 | 354 |
| Old Age, | 188 | 226 | 185 | 186 | 192 |
| Small Pox, | 100 | 152 | 40 | 216 | 426 |

